# Adjustment for Social Risk Factors in a Measure of Clinician Quality Assessing Acute Admissions for Patients With Multiple Chronic Conditions

**DOI:** 10.1001/jamahealthforum.2023.0081

**Published:** 2023-03-10

**Authors:** Kasia J. Lipska, Faseeha K. Altaf, Andrea G. B. Barthel, Erica S. Spatz, Zhenqiu Lin, Jeph Herrin, Susannah M. Bernheim, Elizabeth E. Drye

**Affiliations:** 1 Section of Endocrinology, Department of Medicine, Yale School of Medicine, New Haven, Connecticut; 2 Center for Outcomes Research and Evaluation, Yale New Haven Hospital, New Haven, Connecticut; 3 Now with Genesis Research, Hoboken, New Jersey; 4 Section of Cardiology, Department of Medicine, Yale School of Medicine, New Haven, Connecticut; 5 Section of General Internal Medicine, Department of Medicine, Yale New Haven Hospital, New Haven, Connecticut; 6 Now with Centers for Medicare & Medicaid Services, Baltimore, Maryland; 7 Now with National Quality Forum, Washington, DC

## Abstract

**Importance:**

Adjusting quality measures used in pay-for-performance programs for social risk factors remains controversial.

**Objective:**

To illustrate a structured, transparent approach to decision-making about adjustment for social risk factors for a measure of clinician quality that assesses acute admissions for patients with multiple chronic conditions (MCCs).

**Design, Setting, and Participants:**

This retrospective cohort study used 2017 and 2018 Medicare administrative claims and enrollment data, 2013 to 2017 American Community Survey data, and 2018 and 2019 Area Health Resource Files. Patients were Medicare fee-for-service beneficiaries 65 years or older with at least 2 of 9 chronic conditions (acute myocardial infarction, Alzheimer disease/dementia, atrial fibrillation, chronic kidney disease, chronic obstructive pulmonary disease or asthma, depression, diabetes, heart failure, and stroke/transient ischemic attack). Patients were attributed to clinicians in the Merit-Based Incentive Payment System (MIPS; primary health care professionals or specialists) using a visit-based attribution algorithm. Analyses were conducted between September 30, 2017, and August 30, 2020.

**Exposures:**

Social risk factors included low Agency for Healthcare Research and Quality Socioeconomic Status Index, low physician-specialist density, and Medicare-Medicaid dual eligibility.

**Main Outcomes and Measures:**

Number of acute unplanned hospital admissions per 100 person-years at risk for admission. Measure scores were calculated for MIPS clinicians with at least 18 patients with MCCs assigned to them.

**Results:**

There were 4 659 922 patients with MCCs (mean [SD] age, 79.0 [8.0] years; 42.5% male) assigned to 58 435 MIPS clinicians. The median (IQR) risk-standardized measure score was 38.9 (34.9-43.6) per 100 person-years. Social risk factors of low Agency for Healthcare Research and Quality Socioeconomic Status Index, low physician-specialist density, and Medicare-Medicaid dual eligibility were significantly associated with the risk of hospitalization in the univariate models (relative risk [RR], 1.14 [95% CI, 1.13-1.14], RR, 1.05 [95% CI, 1.04-1.06], and RR, 1.44 [95% CI, 1.43-1.45], respectively), but the association was attenuated in adjusted models (RR, 1.11 [95% CI 1.11-1.12] for dual eligibility). Across MIPS clinicians caring for variable proportions of dual-eligible patients with MCCs (quartile 1, 0%-3.1%; quartile 2, >3.1%-9.5%; quartile 3, >9.5%-24.5%, and quartile 4, >24.5%-100%), median measure scores per quartile were 37.4, 38.6, 40.0, and 39.8 per 100 person-years, respectively. Balancing conceptual considerations, empirical findings, programmatic structure, and stakeholder input, the Centers for Medicare & Medicaid Services decided to adjust the final model for the 2 area-level social risk factors but not dual Medicare-Medicaid eligibility.

**Conclusions and Relevance:**

This cohort study demonstrated that adjustment for social risk factors in outcome measures requires weighing high-stake, competing concerns. A structured approach that includes evaluation of conceptual and contextual factors, as well as empirical findings, with active engagement of stakeholders can be used to make decisions about social risk factor adjustment.

## Key Points

**Question:**

How can policy makers decide when to adjust quality measures for social risk factors?

**Findings:**

In this cohort study of 4 659 922 patients with multiple chronic conditions assigned to 58 435 clinicians, a structured approach that included active engagement of stakeholders, an evaluation of conceptual and contextual factors, as well as empirical analyses of the association of social risk factors with measure scores all led to a consensus to adjust an existing measure for 2 social risk factors.

**Meaning:**

Adjusting measures for social risk factors is controversial, especially in the context of performance measures that influence payment; however, a systematic approach can lead to consensus decisions and more equitable measures.

## Teaser

This cohort study shows a structured, transparent approach to decision-making about adjustment for social risk factors for a measure of clinician quality that assesses acute admissions for patients with multiple chronic conditions.

## Introduction

Whether quality measures should account for differences between clinicians who disproportionately care for beneficiaries with social risk factors—people for whom factors such as income, housing, social support, transportation, and nutrition might adversely affect access to health services or desired health outcomes—remains controversial.^1,2,3,4^ In general, quality measures are risk adjusted for 2 related reasons. The first is to arrive at the most accurate, fair, and unbiased assessment of quality, particularly for comparative purposes. The second is to incentivize behavior in specific directions, usually through financial rewards and punishments. In 2021, Nerenz et al proposed that to assure fairness for clinicians, adjusting for social risk should be the “default option” for measures when empirical arguments for and against adjustment are both valid.^5^ In contrast, Jaffery and Safran argued that outcome measures should never be adjusted for social risk given that this can perpetuate disparities.^6^ Rather, adjustments in payment should be made to promote fairness for clinicians.^6^ Statistically, adjusting for social risk factors sets different expected measure scores for different patient groups (eg, higher expected admission rates for patients with social risk factors). This approach can mask quality differences associated with the social risk factors and perpetuate inequalities that clinicians could potentially address. When used in national programs, adjustment can effectively set differential results based on sociodemographic factors as an expected outcome of US health care. As a result, in its report to Congress, the Office of the Assistant Secretary for Planning and Evaluation specifically recommended that quality measures should not be adjusted for social risk for public reporting.^7^

We confronted the issue of social risk factor adjustment during the development of an outcome quality measure for the Merit-Based Incentive Payment System (MIPS), which makes positive and negative payment adjustments based on ambulatory care quality measures. In this article, we illustrate a structured, transparent approach to decision-making that we used for 1 measure of MIPS clinician quality that assesses acute admissions for patients with multiple chronic conditions (MCCs). Consistent with the guidance drafted by the National Quality Forum (NQF),^8^ we articulated a conceptual model of how social risk factors may influence the specific outcome in the measured patient and clinician populations,^8^ empirically evaluated these associations and the effects of adjustment for social risk factors on measure scores, and considered how the adjusted and unadjusted scores might affect clinician payments given the MIPS program payment structure. Throughout this process, we engaged with stakeholders to help evaluate the conceptual model, empirical findings, and specific programmatic context in which the measure would be used. We present this approach to drive consensus on how to best balance competing concerns of adjustment for social risk factors in other measures.

## Methods

### Data

Under contract with the Centers for Medicare & Medicaid Services (CMS), we developed a MIPS outcome measure for patients with MCCs. We used Medicare administrative claims and enrollment data from calendar years 2017 and 2018, downloaded from the Integrated Data Repository. To identify MIPS-eligible clinicians, we used the 2018 MIPS eligibility file from the CMS Quality Payment Program contractor. To define and identify social risk factors, we used 2013 to 2017 American Community Survey data and 2018 and 2019 Area Health Resource Files.

Research approval was obtained by the Yale University institutional review board, which granted exemption from obtaining patient consent because all patient data were deidentified. This study followed the Strengthening the Reporting of Observational Studies in Epidemiology (STROBE) reporting guidelines.

### Outcome

The measure outcome was the number of all acute unplanned hospital admissions per 100 person-years at risk for admission. The measure did not count planned admissions or admissions for complications of surgeries, accidents, or injuries; admissions directly from a skilled nursing facility (SNF) or acute rehabilitation facility; admissions that occurred within 10 days of discharge from a hospital, SNF, or acute rehabilitation facility; admissions that occurred while patients were enrolled in Medicare’s hospice benefit; and admissions that occurred prior to the first visit in the measurement year with a clinician in the assigned clinician group who had not seen the patient in the prior year. Patients were at risk for the outcome of admission on days they were alive and not in the hospital during the measurement period. In addition to time spent in the hospital, we also excluded from at-risk time: (1) time spent in a SNF or acute rehabilitation facility; (2) the time within 10 days following discharge from a hospital, SNF, or acute rehabilitation facility; (3) time after entering hospice care; and (4) time prior to the patient’s first visit with the attributed clinician unless they were seen by the clinician in the year prior to performance year.

### Cohort and Attribution

The measure cohort included Medicare fee-for-service beneficiaries 65 years or older, alive and not in hospice at the start of the measurement year (2018) and with continuous Medicare Parts A and B enrollment in the measurement year (until death) and year prior (2017), with 2 or more of 9 chronic conditions: (1) acute myocardial infarction; (2) Alzheimer disease and related disorders or senile dementia; (3) atrial fibrillation; (4) chronic kidney disease; (5) chronic obstructive pulmonary disease or asthma; (6) depression; (7) diabetes; (8) heart failure; and (9) stroke or transient ischemic attack.

The measure used a visit-based approach to attribute patients to a primary health care professional or a specialist who typically coordinates or “quarterbacks” care for patients with MCCs (including cardiologists, pulmonologists, nephrologists, neurologists, endocrinologists, and hematologists/oncologists). Patients assigned to each clinician were aggregated at the Taxpayer Identification Number (TIN) level. The attribution algorithm is described in detail in eAppendix 1 in Supplement 1.

### Consideration and Evaluation of Social Risk Factors

#### Stakeholder Engagement

Throughout measure development, we engaged with stakeholders through structured teleconference discussions with a nationally convened technical expert panel (TEP), in-person and virtual meetings with a clinician committee convened by CMS and composed of frontline clinicians and those serving disadvantaged populations, and in writing during a national public comment period. The TEP for this measure was composed of individuals with diverse backgrounds, including clinicians practicing in various settings, patients and caregivers, and other stakeholders with experience in measure development and policy. There were 17 members in the TEP. The Yale Center for Outcomes Research and Evaluation and CMS held meetings with a national TEP throughout the measure development process to review draft measure specifications. Feedback was obtained during these meetings via a round-robin approach along with follow-up discussions. Stakeholders were asked to consider and evaluate the following factors for this measure under MIPS: (1) the conceptual model of how social risk factors may influence admission risk for patients with MCCs; (2) the empirical findings with respect to the strength of the association between social risk factors and admission risk, as well as the association of adjustment for social risk factor with measure scores; and (3) programmatic considerations, including the context in which the measure will be used.

#### Conceptual Model for Risk Adjustment

The overall goal of risk adjustment is to ensure that the measure fairly accounts for patient mix across MIPS clinicians. Hence, we sought to adjust for factors that are associated with the outcome (ie, unplanned hospital admissions), vary across MIPS clinicians, and are unrelated to quality of care, so that measure scores reflect differences in care quality. Inclusion of factors for risk adjustment does not mean that clinicians cannot or should not mitigate their influence on patient outcomes. For example, clinicians can and should treat diabetes, depression, and chronic obstructive pulmonary disease to reduce hospital admissions associated with these factors. The model is designed to reflect their success rather than adjust for it. It adjusts for the presence of chronic conditions at the outset of the measurement period but not for changes in patients’ conditions during the measurement period. The conceptual model for risk adjustment is described in detail in eAppendix 2 in Supplement 1 and illustrated in eFigure 1 in Supplement 1.

### Statistical Analysis

The measure was adjusted for age and 47 clinical and frailty/disability variables (including the 9 cohort-qualifying chronic conditions, other comorbidities, and frailty/disability). Frailty/disability was based on use of durable medical equipment (adapted from a validated claims-based frailty index^9,10^) and original reason for Medicare entitlement. We then evaluated the effect of social risk factor adjustment using social risk factors that (1) could be operationalized in readily available patient-level and area-level data and that (2) we hypothesized MIPS clinicians have the least ability to influence.

The Agency for Healthcare Research and Quality Socioeconomic Status (AHRQ SES) Index summarizes area-level measures of employment, income, education, and housing.^11^ Each of the index components is available at the census block level, which we then used to link to patient’s residence using 9-digit zip codes. Census variables were found in the American Community Survey. We determined low AHRQ SES Index areas by ranking all 9-digit zip codes in the nation and defining the bottom 25th percentile as areas with the lowest AHRQ SES Index.

Low physician-specialist density is a marker of reduced access to specialty care. To calculate this measure, we used Area Health Resources Files to identify the number of physician-specialists, then divided this by the estimated population across areas defined by Federal Information Processing Standards county codes. Low physician-specialist density areas were defined as the bottom 25th percentile of county codes.

Medicare-Medicaid dual-eligibility status is an individual-level marker of low income and assets. Dual eligibility was defined as Medicare patients with full Medicaid benefits for at least 3 months between the measurement year and the second half of the year prior to the measurement year.

We analyzed each of the 3 social risk factors using negative binomial regression modeling for count data. First, we examined the bivariate (unadjusted) association with the outcome. To examine their marginal effect in a risk-adjusted model, we then estimated each variable’s odds ratio in a series of models that adjusted for the measure’s demographic (age), clinical, and frailty/disability (47 variables) factors. We estimated odds ratios for each variable for alternative risk-adjustment strategies. Specifically, we estimated 3 models with only 1 of the 3 social risk factors each (models 1A, 1B, and 1C); 3 models with demographic (age), clinical, and frailty/disability variables and only 1 of the 3 social risk factors (models 2A, 2B, and 2C); a model with low AHRQ SES Index and low physician-specialist density but not Medicare-Medicaid dual eligibility (model 3); and a model with all 3 social risk factors (model 4).

Subsequently, we evaluated the association of caring for dual-eligible patients with MIPS clinicians’ performance by evaluating performance scores (adjusted for the 2 area factors but not dual eligibility) across quartiles of proportion of dual-eligible patients with MCCs. To further assess the sensitivity of the score to adjustment for dual eligibility, we calculated shifts in deciles of measure scores (adjusted for the 2 area factors) with and without dual-eligibility adjustment, as well as the correlation between measure scores with and without dual-eligibility adjustment.

Measure scores were estimated for MIPS clinicians with at least 18 patients with MCCs assigned to them to achieve a minimum measure score reliability of 0.4. Analyses were conducted using SAS, version 9.4 (SAS Institute), and performed between September 30, 2017, and August 30, 2020.

### Program Considerations for Risk Adjustment

To further inform the decision about social risk factor adjustment, we considered how the MIPS program payment formula would use the measure score to create financial incentives for clinicians taking care of patients with social risk factors. At the time of measure development, clinician performance under MIPS was compared with the measure’s benchmark categorized into deciles. Clinicians received 3 to 10 achievement points depending on the decile in which their quality measure score fell. To recognize the challenges and additional costs associated with the care provided to patients with complex medical needs under MIPS, clinicians could earn additional points under the Complex Patient Bonus system. The Complex Patient Bonus was added to the MIPS final score and awarded up to 5 bonus points based on a combination of the average hierarchical condition category risk score of the beneficiaries and the proportion of dually eligible patients treated. Therefore, the Complex Patient Bonus accounts—at least in part—for social risk factors captured by the hierarchical condition category risk score and for the dual-eligible status of patients attributed to MIPS clinicians.

## Results

### Social Risk Factor Adjustment Analyses

There were 4 659 922 patients with MCCs (mean [SD] age, 79.0 [8.0] years; 42.5% male) assigned to 58 435 MIPS clinicians (TINs). Among these patients, more than half had chronic kidney disease and diabetes, while 18.2% resided in areas with a low AHRQ SES Index, 3.6% in areas of low physician-specialist density, and 16.7% were Medicaid-Medicare dual eligible (Table 1).

**Table 1.** Characteristics of Patients Included in the MIPS MCC Measure (N = 4 659 922)

Characteristic	Prevalence, No. (%)
Age, y	
<70	740 962 (15.9)
70 to <75	1 033 292 (22.2)
75 to <80	966 205 (20.7)
80 to <85	823 759 (17.7)
≥85	1 095 704 (23.5)
Chronic disease	
Acute myocardial infarction	100 719 (2.2)
Alzheimer disease and related disorders	1 279 891 (27.5)
Atrial fibrillation	1 167 393 (25.1)
Chronic kidney disease	2 383 858 (51.2)
COPD/asthma	1 613 996 (34.6)
Depression	1 685 967 (36.2)
Heart failure	1 823 667 (39.1)
Stroke/transient ischemic attack	635 160 (13.6)
Diabetes	2 717 638 (58.3)
Frailty indicators^a^	
Walking aid	231 405 (5.0)
Wheelchair	193 552 (4.2)
Hospital bed	75 885 (1.6)
Lift	17 136 (0.4)
Oxygen	383 219 (8.2)
Original reason for entitlement	
DIB (may or may not have ESKD)	685 924 (14.7)
ESKD (may or may not have DIB)	19 072 (0.4)
Social risk factors	
Low AHRQ SES Index (≤25th percentile)	847 802 (18.2)
Low physician-specialist density (≤25th percentile)	167 684 (3.6)
Dual eligibility with ≥3 mo of full Medicaid benefits	776 314 (16.7)

Abbreviations: AHRQ SES, Agency for Healthcare Research and Quality Socioeconomic Status; COPD, chronic obstructive pulmonary disease; DIB, disability insurance benefits; ESKD, end-stage kidney disease; MCC, multiple chronic conditions; MIPS, Merit-Based Incentive Payment System.

^a^ Defined using Noridian policy groups for durable medical equipment or original reason for Medicare entitlement.

The crude unplanned admission rate was 39.1 per 100 person-years. When applying the final model, among the 58 435 MIPS clinicians, the median (IQR) risk-standardized measure score was 38.7 (36.5-41.8) admissions per 100 person-years. Among 31 684 MIPS clinicians (TINs) with a minimum measure score reliability of 0.4 (corresponding to ≥18 patients with MCCs per clinician), the median (IQR) score was 38.9 (34.9-43.6) per 100 person-years.

The distribution of social risk factors across the 31 684 MIPS clinicians (TINs) is shown in eFigure 2 in Supplement 1. The median (IQR) proportion of patients with low AHRQ SES Index across MIPS clinicians was 14.9% (5.6%-30.1%), ranging from 0% to 100%, while the median (IQR) proportion of patients residing in low physician-specialist density areas was 0% (0%-1.4%), ranging from 0% to 100%. Across these MIPS clinicians, the proportion of patients with dual eligibility ranged from 0% to 100% with a median (IQR) of 9.5% (3.1%-24.5%).

Low AHRQ SES Index and low physician-specialist density were significantly but weakly associated with the risk of hospitalization in the univariate models (relative risk [RR], 1.14; 95% CI, 1.13-1.14, and RR, 1.05; 95% CI, 1.04-1.06, respectively), while Medicare-Medicaid dual eligibility was more strongly associated (RR, 1.44; 95% CI, 1.43-1.45) (Table 2). However, the effect of dual-eligibility status was attenuated by adjustment for the demographic, clinical, and frailty/disability characteristics (RR, 1.11; 95% CI, 1.11-1.12) and relatively unchanged when the other 2 social risk factors were also in the model (RR, 1.10; 95% CI, 1.10-1.11).

**Table 2.** Association of Social Risk Factors With Hospitalization Rates in Univariate and Multivariate Analyses

Social risk factor	Relative risk (95% CI)			
	Univariate models 1A, 1B, and 1C (unadjusted)	Multivariate models 2A, 2B, and 2C (adjusted for age and 47 clinical and frailty/disability variables)	Multivariate model 3 (model 2 + low AHRQ SES Index and low physician-specialist density)	Multivariate model 4 (model 3 + Medicare-Medicaid dual-eligibility status)
Low AHRQ SES Index	1.14 (1.13-1.14)	1.08 (1.07-1.08)	1.08 (1.07-1.08)	1.06 (1.06-1.07)
Low physician-specialist density	1.05 (1.04-1.06)	1.05 (1.04-1.06)	1.04 (1.03-1.05)	1.04 (1.03-1.05)
Medicare-Medicaid dual-eligibility status	1.44 (1.43-1.45)	1.11 (1.11-1.12)	NA	1.10 (1.10-1.11)

Abbreviations: AHRQ SES, Agency for Healthcare Research and Quality Socio-economic Status; NA, not applicable.

**Figure 1.**
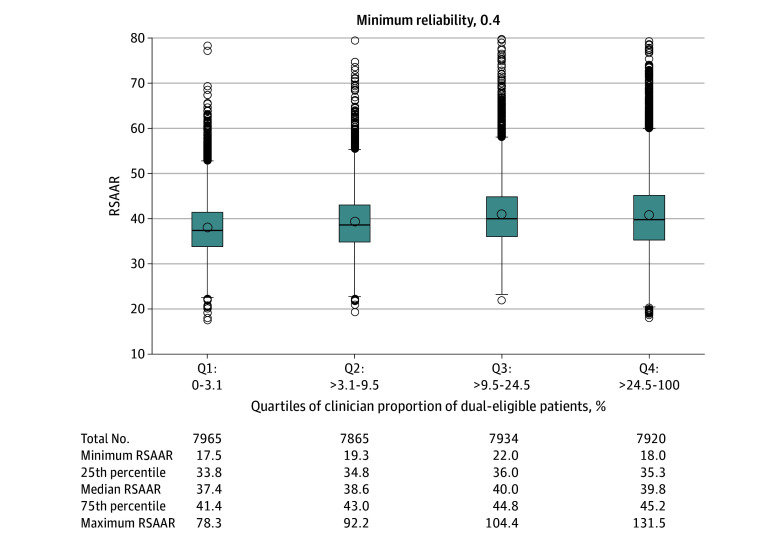
Distribution of Measure Scores by Quartiles of Medicare-Medicaid Dual-Eligible Patients The minimum measure score reliability was 0.4. Box plots show mean score (circle inside each box), median score (line inside each box), IQR (bottom and top edges of each box), and 1.5 × IQR (error bars). RSAAR indicates risk-standardized acute admission rate.

One option CMS considered was adjusting for low AHRQ SES Index and low physician-specialist density but not for dual-eligibility status. To illuminate the implications of this option, risk-standardized hospital admission rates across MIPS clinicians caring for variable proportions of dual-eligible patients with MCCs were evaluated (quartile 1, 0%-3.1%; quartile 2, >3.1%-9.5%; quartile 3, >9.5%-24.5%, and quartile 4, >24.5%-100%) (Figure 1). The median measure scores were 37.4, 38.6, 40.0, and 39.8 per 100 person-years for quartiles 1 through 4, respectively, with large overlaps in performance across the 4 groups.

**Figure 2.**
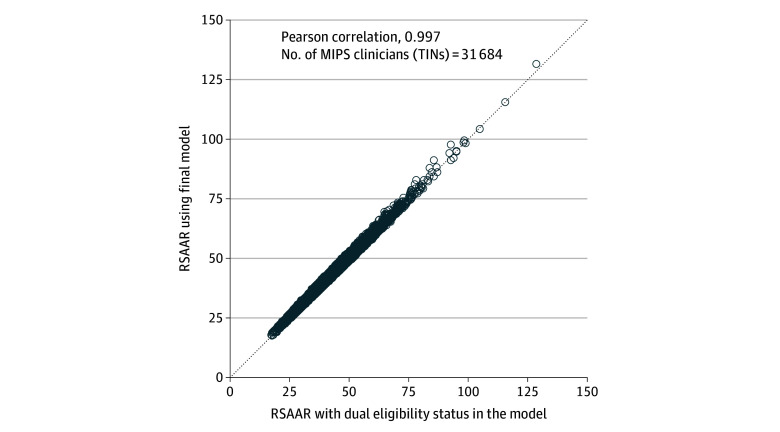
Risk-Standardized Acute Admission Rates (RSAARs) With and Without Inclusion of Medicare-Medicaid Dual-Eligibility Status in the Models The minimum measure score reliability was 0.4 (≥18 patients with multiple chronic conditions). MIPS indicates Merit-Based Incentive Payment System; TINs, Taxpayer Identification Numbers.

If the measure was also adjusted for dual-eligibility status, 14.4% of MIPS clinicians’ measure scores would change performance ranking by a decile, while 0.1% would change performance ranking by 2 deciles. The Pearson correlation between risk-standardized admission rates with and without inclusion of dual eligibility in the model was strong at 0.997 (Figure 2).

### Stakeholder Input

Given that in the MIPS program accountability lies with clinicians or clinician groups who may have a more limited ability to mitigate the effect of social risk factors on outcomes compared with larger organizations, such as accountable care organizations, stakeholders generally supported the inclusion of the 2 area-level factors in the final risk-adjustment model. However, they were more divided on dual eligibility. Some TEP members stated that the AHRQ SES Index is a preferred variable relative to dual eligibility because the AHRQ SES Index is derived from a national data source, which makes it valid for drawing comparisons on clinician performance; this is in contrast with the measure of dual eligibility that is specific to a clinician’s geographic location, especially state. Furthermore, clinicians may be able to mitigate the risk associated with dual eligibility, especially for patients who do not reside in resource-deprived neighborhoods. Stakeholders also considered the empirical findings of the attenuated association of dual eligibility with the outcome in a model adjusted for AHRQ SES Index. Finally, the existence of the Complex Patient Bonus, which takes into account the proportion of dual-eligible patients treated by MIPS clinicians, further mitigated concerns that a measure unadjusted for dual eligibility would disadvantage these clinicians.

### Final Model

Balancing these conceptual considerations, empirical findings, and programmatic structure, CMS decided to adjust the MIPS MCC admission measure for the AHRQ SES Index and the physician-specialist density (ie, the 2 area-level indicators of social risk) but not for the dual-eligibility status of patients. The NQF committees subsequently voted to endorse the measure for use.

## Discussion

This analysis illustrates how conceptual and contextual considerations, as well as empirical data, can inform a transparent decision-making process about whether to adjust quality measures for social risk factors. We explicitly laid out our assumptions about clinicians’ influence on social risk factors, shared the empirical findings showing the influence of social factor adjustment on model performance and the measure scores with stakeholders, and considered program design and context in these considerations. Using this approach, we made transparent the information that CMS used to ultimately make the decision on social risk factor adjustment. The final MIPS MCC measure was adjusted for 2 social risk factors, the AHRQ SES Index and physician-specialist density, but not for dual-eligibility status, to account for area factors that could not be practically mitigated by physicians who care for the patients.

Measure developers can use this approach to systematically consider conceptual and programmatic factors and empirical findings to guide decisions about social risk factor adjustment. In this analysis, MIPS MCC measure scores changed minimally after adjustment for dual-eligibility status—potentially providing reassurance to clinicians who may feel disadvantaged by a measure unadjusted for this risk factor. The stronger effect of adjustment with performance seen in prior studies may be due to less complete adjustment for clinical and functional status factors in the measures studied.^12^ In addition, we found that some clinicians caring for a high proportion of dual-eligible patients performed very well on the measure, consistent with the conceptual model and evidence demonstrating that dual-eligible patients’ increased risk of admission can be successfully addressed through provision of high-quality care. Future work should explore factors associated with success so that best practices may be shared across clinician networks.

Programmatic context is critical to considerations about social risk factor adjustment. Rather than adjusting measures to avoid payment adjustment, programs can and do provide additional bonuses to safety-net clinicians who care for patients with complex medical needs. When these programs do exist, they may obviate the need for adjustment.^6^ Notably, more work needs to be completed to understand the success of program-level incentives; for example, one study found that the Complex Patient Bonus, as currently designed, had no substantive influence on the association between treating a larger number of socially at-risk Medicare beneficiaries and reduced MIPS reimbursement.^13^ Ultimately, considerations about social risk factor adjustment require developing and evaluating conceptual and statistical models in the context of the program in which the measure will be used. This approach is aligned with new recommendations from the NQF that require measure developers to present a conceptual model and consider a measure’s intended use.^14^ However, the NQF has not yet formally integrated this approach into its measure-endorsement criteria.

The approach we propose requires obtaining and incorporating stakeholder input from multiple perspectives, including those of patients, clinicians, measured entities, health care organizations, payers, professional societies, and measure experts. Effective stakeholder engagement requires substantial commitment, and facilitated meetings can be costly; in-person meetings fostered understanding, but remote gatherings could be substituted.

### Limitations

This study has several limitations. For the empirical findings, we used well-established definitions for low AHRQ SES Index, low physician-specialist density, and Medicare-Medicaid dual eligibility, but we could not capture other facets of social risk in our evaluations. Because some of the observed associations between social risk factors and performance on quality measures may be the result of underlying differences in medical complexity, frailty, disability, and/or functional status, it is important to consider these factors in risk adjustment. We adjusted for frailty/disability using durable medical equipment variables adapted from a validated claims-based frailty index; however, we did not use the validated index itself. The present data included only Medicare fee-for-service beneficiaries cared for by MIPS-eligible clinicians. Whether these findings would be similar for Medicare Advantage, commercially insured, or younger patients with MCCs is unknown. We were unable to quantitatively evaluate how the Complex Patient Bonus affected payment adjustments. Yet, we provide a case example of navigating competing concerns in quality measurement; future studies will need to test whether this approach can drive consensus in other contexts.

## Conclusions

This cohort study demonstrated that adjustment for social risk factors in outcome measures requires weighing high-stakes, competing concerns. We provided an illustrative example of how conceptual and contextual factors, quantitative analyses, and evaluation by stakeholders of these factors can be used to make decisions about social risk factor adjustment. This approach requires (1) stakeholder engagement to guide evaluation of concepts and analytic results and a careful consideration of the pros and cons of risk adjustment through a transparent process; (2) a conceptual model that illuminates how social risk factors may affect the outcome and how clinicians in the intended accountability program may mitigate these effects; (3) empirical findings that quantify the influence of social risk factor adjustment; and (4) considerations of the program payment context. This approach is relatively resource intensive, requiring iterative consultation with stakeholder groups, and may not always be feasible. However, it holds the promise of widening health care professionals’, patients’, and other stakeholders’ acceptance of a measure as valid and actionable, and therefore potentially furthers its use and, ultimately, its effect on improving quality.

## Notes

Supplement 1. **eFigure 1.** Conceptual model for risk factor adjustment in the MIPS admission measure for patients with multiple chronic conditions **eAppendix 1.** Attribution algorithm **eAppendix 2.** Conceptual model for risk adjustment **eFigure 2.** Distribution of social risk factors among 31,684 MIPS clinicians and clinician groups

Supplement 2. Data Sharing Statement
