# Supplementary material for: Adjustment for Social Risk Factors in a Measure of Clinician Quality Assessing Acute Admissions for Patients With Multiple Chronic Conditions
Source: JAMA Health Forum. 2023 Mar 10;4(3):e230081. doi: 10.1001/jamahealthforum.2023.0081 (PMC12124487; doi:10.1001/jamahealthforum.2023.0081)
Supplement: Supplement 1. — eFigure 1. Conceptual model for risk factor adjustment in the MIPS admission measure for patients with multiple chronic conditions eAppendix 1. Attribution algorithm eAppendix 2. Conceptual model for risk adjustment eFigure 2. Distribution of social risk factors among 31,684 MIPS clinicians and clinician groups [file jamahealthforum-e230081-s001.pdf]

Supplemental Online Content

Lipska KJ, Altaf FK, Barthel AGB, et al. Adjustment for social risk factors in a measure of clinician quality assessing acute admissions for patients with multiple chronic conditions. *JAMA Health Forum*. 2023;4(3):e230081.
doi:10.1001/jamahealthforum.2023.0081

eFigure 1. Conceptual model for risk factor adjustment in the MIPS admission measure for patients with multiple chronic conditions
eAppendix 1. Attribution algorithm
eAppendix 2. Conceptual model for risk adjustment
eFigure 2. Distribution of social risk factors among 31,684 MIPS clinicians and clinician groups

This supplemental material has been provided by the authors to give readers additional information about their work.

eFigure 1. Conceptual model for risk factor adjustment in the MIPS admission measure for patients with multiple chronic conditions

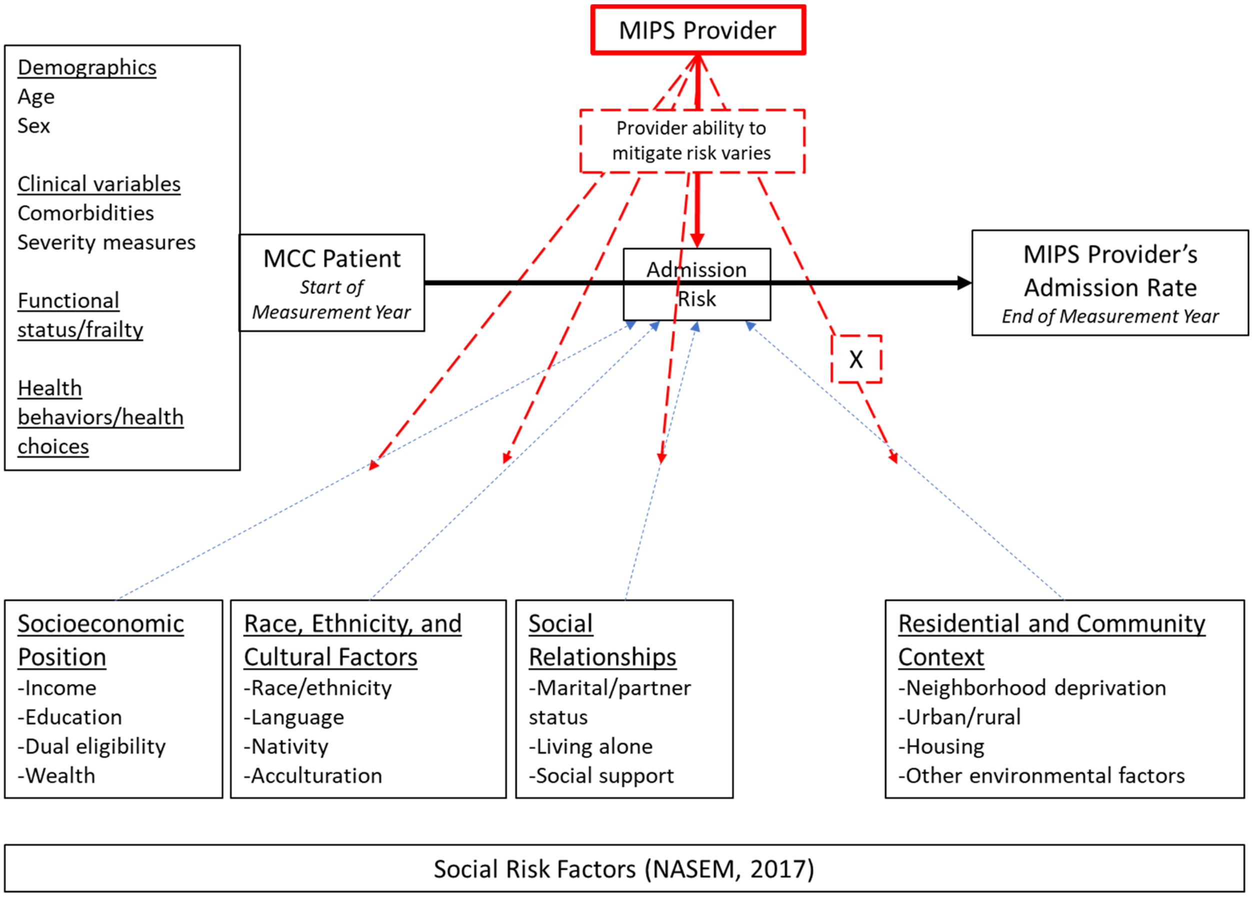

© 2023 Lipska KJ et al. *JAMA Health Forum.*

eAppendix 1. Attribution algorithm

The attribution algorithm first assigned patients to the clinician (based on NPI/TIN) most responsible for the patient's care. Patients were assigned to the PCP at or over the 2-visit minimum threshold. However, the PCP assignment was overridden if the patient had at least 2 more visits with a relevant specialist than with any other clinician (PCP or other specialist), in which case the patient was assigned to that "dominant" specialist. Patients who were ultimately assigned to a hematologist/oncologist were excluded from the measure since oncologic care predominates for these patients. In a second step, patients assigned to each NPI/TIN were aggregated at the TIN level, the level of the MIPS measure score, for TINs with multiple NPIs. That is, patients "followed" their assigned clinician (NPI/TIN) to the TIN.

© 2023 Lipska KJ et al. *JAMA Health Forum.*

eAppendix 2. Conceptual model for risk adjustment

We identified potential candidate risk factors hypothesized to affect the outcome through: (1) prior work on related quality measures (specifically, the MCC measure previously developed for ACOs), (2) a focused literature review, and (3) Technical Expert Panel (TEP), Clinician Committee, and public input. Adapting a conceptual model we had developed with CMS for the ACO MCC admission measure,[9] we defined and illustrated the potential relationships between different categories of risk factors and the outcome of hospital admissions (see Appendix Figure 1). We identified patient demographic factors and clinical variables, including comorbidities and measures of frailty and disability, that reflect the characteristics of the patients at the start of the measurement year.

To conceptualize and categorize social risk factors, we adopted the framework of the National Academies of Sciences, Engineering, and Medicine (NASEM) comprehensive, expert report of 2017, in which they categorized social risk factors into the four domains represented in the bottom row of Figure 1:[10]

- Socioeconomic position;
- Race, ethnicity, and cultural factors;
- Social relationships; and
- Residential and community context.

(Note: There is a fifth domain in the NASEM report related to gender and sexual orientation; however, we left it out because the authors noted that more research is needed to understand the relationship of these factors to outcomes and because of lack of available data.)

Variables in all of these domains are hypothesized to be associated with increased risk of admission. The domains differ, however, in the extent to which we expect an individual MIPS clinician or group of clinicians to be able to mitigate the risk conferred by such variables. These differences inform their

potential use as risk adjusters in this specific MIPS measure, since adjusting for factors that can more easily be mitigated by higher quality care is more likely to mask low quality care.

As represented by a boxed “X” in the conceptual model figure, MIPS providers have the least ability to mitigate the risk of admission associated with broader residential and community factors, such as neighborhood deprivation and relative lack of access to primary and specialty medical care. In contrast, however, we expect that there is more (although not unlimited) ability for a MIPS provider to intervene to mitigate some or all of the risk conferred by the other, individual-level domains noted above. For example, a provider can take into account a patient’s education level, health literacy level, and home living situation when planning and delivering care. In addition, high-quality care may be characterized as being more racially, linguistically, and culturally sensitive and informed. While such tailored care can likely mitigate risk of admission, our TEP emphasized that providing it also requires resources so MIPS providers may be limited in their capacity to deliver it.

© 2023 Lipska KJ et al. *JAMA Health Forum.*

eFigure 2. Distribution of social risk factors among 31,684 MIPS clinicians and clinician groups

A) Low AHRQ SES index, B) Low physician-specialist density, C) Medicare-Medicaid dual eligibility.

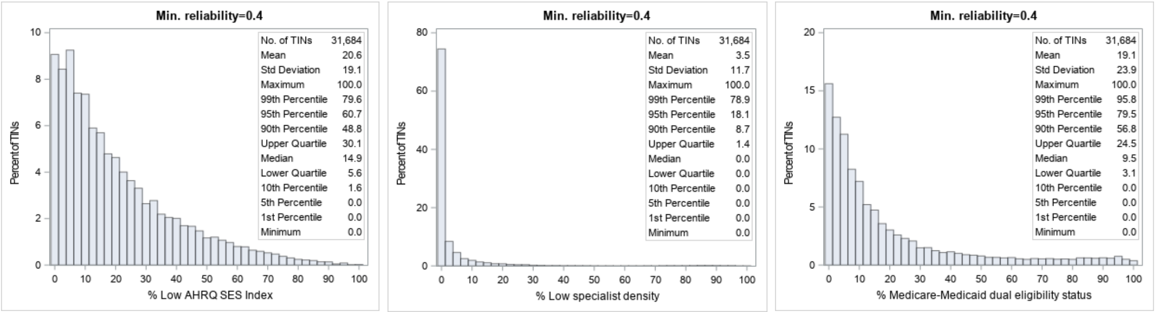

© 2023 Lipska KJ et al. *JAMA Health Forum.*
