# Supplementary material for: Adjustment for Social Risk Factors in a Measure of Clinician Quality Assessing Acute Admissions for Patients With Multiple Chronic Conditions
Source: JAMA Health Forum. 2023 Mar 10;4(3):e230081. doi: 10.1001/jamahealthforum.2023.0081 (PMC12124487; doi:10.1001/jamahealthforum.2023.0081)
Supplement: Supplement 2. — Data Sharing Statement [file jamahealthforum-e230081-s002.pdf]

Data Sharing Statement

Lipska. Adjustment for Social Risk Factors in a Measure of Clinician Quality Assessing Acute Admissions for Patients With Multiple Chronic Conditions. *JAMA Health Forum*. Published March 10, 2023. doi:10.1001/jamahealthforum.2023.0081

Data
Data available: No
